# Prognostic value of soluble ST2 in adults with congenital heart disease

**DOI:** 10.1136/heartjnl-2018-314168

**Published:** 2019-01-30

**Authors:** Laurie W Geenen, Vivan J M Baggen, Annemien E van den Bosch, Jannet A Eindhoven, Judith A A E Cuypers, Maarten Witsenburg, Eric Boersma, Jolien W Roos-Hesselink

**Affiliations:** 1 Department of Cardiology, Erasmus MC, University Medical Centre Rotterdam, Rotterdam, The Netherlands; 2 Department of Clinical Epidemiology, Erasmus MC, University Medical Centre Rotterdam, Rotterdam, The Netherlands

**Keywords:** congenital heart disease

## Abstract

**Objective:**

Soluble suppression of tumourigenicity-2 (sST2) is upregulated as response to myocardial stress and may be a potential biomarker for risk stratification in patients with adult congenital heart disease (ACHD). This study aimed to investigate the release of sST2 and its association with cardiovascular events in ACHD.

**Methods:**

In this prospective cohort study, 602 consecutive patients with ACHD visiting the outpatient clinic were included (2011–2013). The association between sST2 and a primary composite endpoint of all-cause mortality, heart failure, hospitalisation, arrhythmia, thromboembolic events or cardiac interventions was investigated using multivariable Cox regression.

**Results:**

sST2 was measured in 590 (98%) patients (median age 33 [25–41] years, 42% women). After a median follow-up of 5.8 [IQR 5.1–6.2) years, 225 (38.5%) reached the primary endpoint. sST2 was significantly associated with the primary endpoint when adjusted for age, sex, creatinine and N  terminal pro-B type brain natriuretic peptide (NT-proBNP) (HR per twofold higher sST2: 1.28, 95% CI 1.03 to 1.58, p=0.025). This association negated when adjusted for clinical variables and NT-proBNP (HR per twofold higher sST2: 1.19, 95% CI 0.96 to 1.48, p=0.106). Stratified analysis in complex ACHD did show a significant association between sST2 and the primary endpoint when adjusted for clinical variables and NT-proBNP (HR per twofold higher sST2: 1.31, 95% CI 1.01 to 1.69, p=0.043). Sex-specific analysis showed an association between sST2 and the primary endpoint in women (HR per twofold higher sST2 1.80, 95% CI 1.30 to 2.49, p<0.001) but not in men (HR per twofold higher sST2 1.19, 95% CI 0.90 to 1.56, p=0.223).

**Conclusions:**

sST2 is a promising novel biomarker in patients with ACHD, specifically in complex ACHD and women. Future research is warranted to elucidate sex-specific and diagnosis-specific differences.

## Introduction

With the improvement of genomic technology, new pathways and biomarkers related to cardiac remodelling are discovered. One of these recently discovered biomarkers is soluble suppression of tumourigenicity-2 (sST2), a member of the interleukin-1 receptor family. The expression of sST2 is upregulated by cardiac myocytes as a response to stress or injury.^1^ Higher sST2 levels are found in patients with severe heart failure (HF) and are associated with an increased mortality.^2^ sST2 is therefore seen as a promising new biomarker in the ongoing search for the ideal HF biomarker for optimal risk stratification. Adequate risk stratification can contribute to better individualised therapeutic strategies, improving survival and reducing morbidity. Additionally, it can contribute to more detailed tailoring of information about the prognosis of an individual patient.

While sST2 has been extensively investigated in several disease populations such as patients with myocardial infarction^3 4^ and chronic and acute HF,^2 5 6^ only very limited data are available in adult congenital heart disease (ACHD).^7^ The ACHD population is rapidly expanding because of improved therapies during childhood and are characterised by a high burden of HF, arrhythmias and (re-)interventions at adult age.^8^ Proper risk stratification is therefore of major importance. It is unknown whether sST2 is associated with more complex ACHD and whether it yields additive prognostic value. Therefore, this study aimed to investigate the release of sST2 among different types of ACHD and its association with cardiovascular events in patients with moderate and complex ACHD, beyond the conventional biomarker N terminal pro-B type brain natriuretic peptide (NT-proBNP).

## Methods

### Study population and healthy controls

In this prospective observational cohort study, consecutive adults patients with moderate or complex^9^ congenital heart disease who routinely visited the outpatient clinic of our centre between April 2011 and April 2013 were included. Exclusion criteria were: age <18 years, pregnancy, mild cardiac lesion (isolated atrial or ventricular septal defect), severe renal dysfunction (creatinine >200 µmol/L) or not capable of understanding or signing informed consent. At the day of study inclusion, all patients underwent physical examination by a cardiologist, 12-lead electrocardiography, transthoracic echocardiography and venous blood sampling. Follow-up was ensured by protocolled structural annual visits to the ACHD outpatient clinic during the first four subsequent years. The study protocol conforms to the principles outlined in the Declaration of Helsinki. All participating patients gave written informed consent. The study protocol and echocardiographic imaging analysis have been described in more detail previously.^10 11^

A healthy control cohort consisting of self-declared healthy volunteers was recruited between January 2014 and December 2014. All volunteers underwent physical examination, electrocardiography, echocardiography and venous blood sampling on the same day. More details have been described previously.^12^

### Biomarker assessment

Venous blood samples were taken at the day of study inclusion for study purpose only. No clinical decisions were based on any biomarker. Blood samples were transferred to the clinical chemistry laboratory within 2 hours. NT-proBNP and creatinine were directly determined in fresh blood samples. The rest of the samples were aliquoted and stored at −80°C until batch analysis was performed. Serum sST2 was measured on the Presage ST2 assay (Critical Diagnostics, San Diego, California, USA), a monoclonal electrochemiluminescense immunoassay (limit of quantitation 2.4 ng/mL). Samples were exposed to two thaw-freeze cycles before analysed. The Presage ST2 assay is not significantly affected by sample freeze–thaw cycles and is stable up to 15 freeze–thaw cycles. sST2 was measured once in study patients and twice in healthy volunteers, in order to assess reproducibility and to obtain reference values.

### Definition and assessment of study endpoints

The primary endpoint was defined prior to the collection of data as a composite of all adverse cardiovascular events: all-cause mortality, HF (requiring initiation or change in HF medication or requiring hospitalisation), hospitalisation for cardiac reasons, arrhythmia (symptomatic and recorded, or requiring treatment), thromboembolic events (ischaemic cerebrovascular accident, pulmonary embolism or myocardial infarction) or cardiac interventions (surgical or percutaneous). We defined the secondary endpoint as a composite of all-cause mortality or HF. All patients were annually evaluated at our outpatient clinic according to a standard protocol. Information was retrieved from electronic patient records. Survival status was checked in the Municipal Population Register. Ambiguous endpoint events were adjudicated by two investigators (LWG and JWR-H) without knowledge of any biomarker level. All patients who did not reach one of the endpoints were censored after 1 January 2018.

### Statistical analysis

Sample size calculation was performed and has been described previously.^10^ Continuous variables are represented as mean±SD or median (IQR). The X^2^ Mantel-Henszel test for trend or linear regression was performed to compare variables across the different quartiles of sST2. The correlation between sST2 and NT-proBNP was visualised with scatterplots, and the Spearman correlation coefficient was calculated.

Reproducibility of ST2 assay was assessed by Bland-Altman plots with corresponding limits of agreement. The coefficient of variation was determined by the following calculation; SD of the differences of two measurements divided by the mean of two measurements*100%.

The upper limit of normal was determined based on the 97.5th percentile of sST2 levels in healthy volunteers. The 97.5th percentile was estimated using 2log transformed sST2 values and calculated with mean +1.96 SD.^13^ Sex specific reference values were calculated.

We used the Kaplan-Meier method to derive the cumulative endpoint-free survival estimates. Survival curves stratified according to the quartile distribution of sST2 were compared with the log-rank test for trend. Cox proportional hazard regression was performed to assess the association between sST2 and the endpoints. Multivariable analyses were performed to adjust for clinical characteristics, creatinine and NT-proBNP. Additivity of sST2 and sex was performed using an interaction term and tested with the log-likelihood ratio test. Likewise, linearity of sST2 was checked by adding a natural cubic spline with 3 df. Data on NT-proBNP were missing in <1% and were completed by imputation of the mean.

As post hoc analysis, patients were stratified according to moderate and complex congenital heart disease, and the association between sST2 and both endpoints was assessed with Cox regression. Analyses were performed using IBM SPSS Statistics (V.24) and R (V.3.5.1, packages survival). A two-sided p value below 0.05 was considered statistically significant.

## Results

### Baseline characteristics

In 590 of the 602 patients with a moderate to complex ACHD who were originally included in this cohort, sST2 was measured (online supplementary file 1). The median age of the patients was 33 (IQR 25–41) years, 248 (42%) were women and 90% was in New York Heart Association (NYHA) class I (table 1).

10.1136/heartjnl-2018-314168.supp1 Supplementary data

**Table 1:** Baseline characteristics for all patients and stratified according to the quartile distribution of sST2

	All (n=590)	sST2 quartiles				P value for trend
		First <18.0 ng/mL (n=147)	Second 18.0–24.3 ng/mL (n=149)	Third 24.3–32.2 ng/mL (n=149)	Fourth >32.2 ng/mL (n=145)	
**Clinical characteristics**						
Age, years	33 (25-41)	34 (26-43)	33 (25-41)	33 (25-43)	31 (24-37)	0.039
Sex, women, n (%)	248 (42)	100 (68)	80 (54)	45 (30)	23 (16)	<0.001
Surgical repair, n (%)	538 (91)	137 (93)	135 (91)	131 (88)	135 (93)	0.769
Age at surgical repair, years	3.8 (0.8–11.9)	4.4 (1.0–13.9)	3.3 (0.8–11.1)	3.6 (0.7–11.7)	3.3 (0.7–12.2)	0.324
Congenital diagnosis, complex, n (%)*	324 (55)	71 (48)	79 (53)	88 (59)	86 (59)	0.033
Cardiac medication use, n (%)†	211 (36)	55 (37)	48 (32)	56 (38)	52 (36)	0.956
Body mass index, kg/m^2^	24.8±4.4	25.3±4.6	24.6±3.9	24.9±4.7	24.1±4.2	0.040
Heart rate, beats/minute	74±13	74±13	72±14	74±13	75±13	0.332
Systolic blood pressure, mm Hg	126±16	126±19	126±16	126±14	127±16	0.682
O_2_saturation <90%, n (%)	17 (3)	0 (0)	2 (1)	5 (3)	10 (7)	<0.001
NYHA class, II or III, n (%)	61 (10)	10 (7)	10 (7)	18 (12)	23 (16)	0.004
**Electrocardiography**						
Rhythm, n (%)						0.153
Sinus rhythm	509 (86)	121 (82)	131 (88)	135 (91)	122 (84)	
Paced rhythm	44 (8)	13 (9)	10 (7)	6 (4)	15 (10)	
Other	37 (6)	13 (9)	8 (5)	8 (5)	8 (6)	
QRS duration, ms	113 [100-137]	110 [95-128]	112 [99-136]	115 [101-148]	115 [104-138]	0.002
**Echocardiography**						
Left atrial volume, mL/m^2^	21 [16 -29]	20 [16-29]	21 [15-29].5	20 [15-29]	21 [15-30]	0.656
Left ventricular end-diastolic volume, mL/m^2^*^‡^	63±19	60±20	63±18	64±17	66±20	0.019
Left ventricular ejection fraction, %‡	56±8	57±8	56±9	56±7	56±7	0.218
Right ventricular end diastolic annulus, mm	42±8	41±8	41±8	43±8	44±8	0.002
Right ventricular fractional area change, %	38±11	40±12	38±10	37±12	38±11	0.200
Systemic ventricular function, n (%)						0.202
Normal	296 (50)	81 (55)	79 (53)	67 (45)	69 (48)	
Mildly impaired	207 (35)	48 (33)	47 (32)	59 (40)	53 (37)	
Moderately impaired	69 (12)	13 (9)	17 (11)	20 (13)	19 (13)	
Severely impaired	18 (3)	5 (3)	6 (4)	3 (2)	4 (3)	
E/A ratio	1.6±0.7	1.7±0.8	1.6±0.6	1.6±0.6	1.7±0.6	0.910
E′ wave, m/s	8.2±2.6	8.3±2.7	8.1±2.5	8.0±2.5	8.5±2.6	0.011
E/E′ ratio	11.6±5.1	12.1±5.7	11.7±4.1	11.4±5.0	11.2±5.3	0.220
Severe valvular dysfunction, n (%)§	83(14)	16 (11)	21 (14)	21 (14)	25 (18)	0.136
**Laboratory results**						
Creatinine, μmol/L	77±18	74±15	73±13	79±17	82±24	<0.001
NT-proBNP, pmol/L¶	15 [7-33]	18 [8-36]	13 [7-33]	15 [6-30]	15 [6-29]	0.481
sST2, ng/mL	24.3 (18.0–32.2)	14.0 (11.4–16.1)	21.0 (19.5–22.5)	28.1 (26.1–30.0)	39.9 (34.9–49.6)	-

*Congenital diagnosis of arterial switch operation, aortic stenosis or aortic coarctation (0) versus tetralogy of Fallot, Rastelli, systemic right ventricle, univentricular heart or pulmonary arterial hypertension (1).

†Beta-blocker (=90, 15%), ACE inhibitor (n=88, 15%), diuretic (n=71,12%), antiarrhythmic (n=53, 9%) angiotensin receptor blocker (n=36, 6%).

‡Left-sided volumes were not measured in patients with a systemic right ventricle, univentricular heart, pulmonary hypertension or a poor acoustic window.

§Defined as maximal aortic or pulmonary valve velocity >4.0 m/s; grade 3 or 4 out of 4 aortic, pulmonary or mitral valve regurgitation; or grade 4 out of 4 tricuspid valve regurgitation.

¶Analysis was performed based on 2log transformed values.

NT-proBNP, N terminal pro-B type brain natriuretic peptide; NYHA, New York Heart Association; sST2, soluble suppression of tumourigenicity-2.

**Figure 1:**
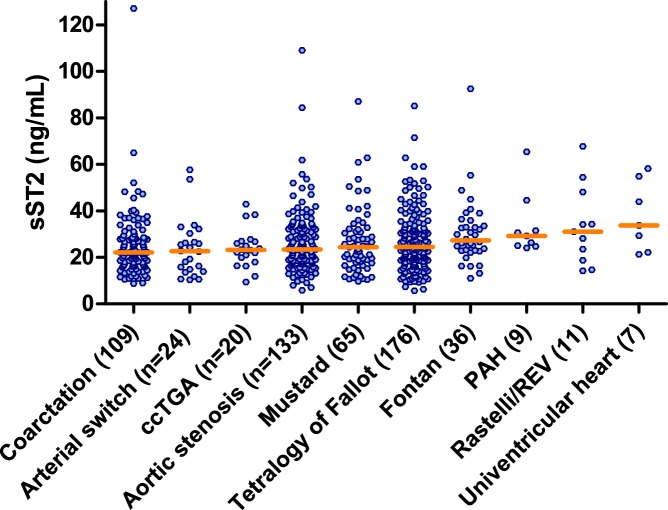
sST2 levels according to the different congenital diagnosis groups. The median sST2 level in each group is indicated by the horizontal line. ccTGA, congenital corrected transposition of the great arteries; PAH, pulmonary arterial hypertension; sST2, soluble suppression of tumourigenicity-2.

Median sST2 levels in women and men were 19.5 (IQR 14.5–25.2) ng/mL and 28.5 (IQR 21.9–36.7) ng/mL, respectively. sST2 was elevated in seven women (2.8%) and 15 men (4.4%). Higher levels of sST2 were associated with oxygen saturation <90%, higher NYHA class, longer QRS duration, higher left ventricular end-diastolic volume and a larger right ventricular end diastolic annulus diameter (table 1). Highest sST2 levels were found in patients with Fontan, pulmonary arterial hypertension, Rastelli/reparation à l’etage ventriculaire and univentricular heart (figure 1). No significant correlation was found between levels of sST2 and NT-proBNP in men, and only a very weak correlation was found in women (r=−0.17, p=0.002). In moderate ACHD, a weak negative correlation was found (r=−0.19, p=0.001) (online supplementary file 2).

### Reference values and reproducibility

**Figure 2:**
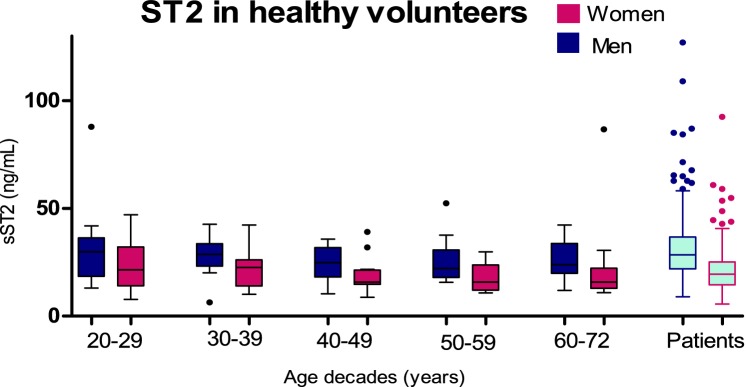
sST2 levels according to age decades and sex in healthy volunteers shown by boxplots. sST2, soluble suppression of tumourigenicity-2.

sST2 was measured in 142 healthy volunteers. One healthy volunteer was excluded from analysis because sST2 measurement differed >20 SDs from the mean sST2 value of the healthy cohort and was seen as extreme outlier. sST2 was significantly higher in men than in women (p=0.002) but not associated with age (♂p=0.138, ♀p=0.334)(figure 2). Sex-specific upper limits of normal of sST2 were 44.50 ng/mL for women and 55.85 ng/mL for men. Percentile levels of sST2 in healthy volunteers and patients with ACHD are summarised in online supplementary file 3. Reproducibility of the sST2 essay was good, with a coefficient of variation of 7.74% and limits of agreement of −5.59 ng/mL to 7.61 ng/mL (online supplementary file 4).

### Follow-up

Survival status was complete in 99.7%. Detailed follow-up data regarding the other endpoints were available in 585 patients (99.2%). After a median of 5.8 (IQR 5.1–6.23) years of follow-up, the primary composite endpoint occurred in 225 patients (38.5%). The secondary endpoint occurred in 69 patients (11.8%). With regard to all separate components of the primary endpoint (ie, patients were not censored at the time of another endpoint than the endpoint of interest), the occurrence of events were: death (n=25), HF (n=59), hospitalisation (n=177), arrhythmia (n=127), thromboembolic event (n=29) and cardiac intervention (n=135).

### sST2 and associations with study endpoints

**Figure 3:**
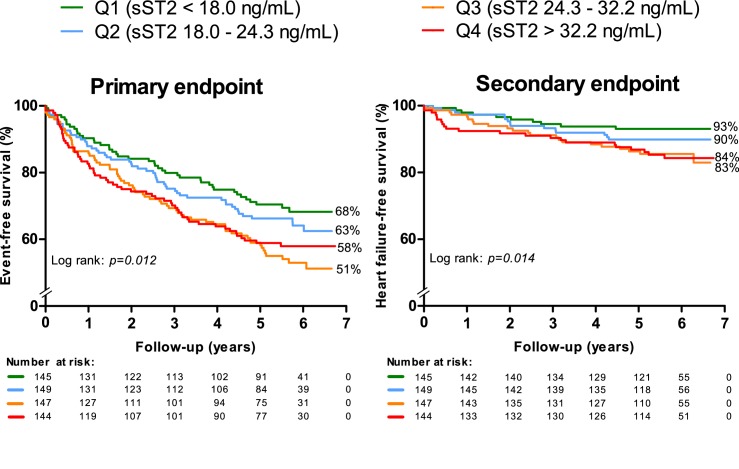
Survival regarding the primary endpoint (any cardiovascular event) and the secondary endpoint (death or heart failure) stratified according to the quartile distribution of sST2. Q1, quartile 1; Q2, quartile 2; Q3, quartile 3; Q4, quartile; sST2, soluble suppression of tumourigenicity-2.

Endpoint-free survival stratified according to the quartile distribution of sST2 showed that patients in the lowest sST2 quartile (Q1; sST2 18.0<ng/mL) had a significant better primary and secondary endpoint-free survival than patients in higher quartiles (figure 3).

Multivariable analysis with continuous sST2 levels and adjustment for age, sex and creatinine showed a significant association between sST2 and both the primary and secondary endpoints (table 2). Additional adjustment for NT-proBNP showed that a twofold increase in sST2 level was significantly associated with an increased risk of both endpoints. sST2 was also independently associated with the endpoints after full adjustment for age, sex and other clinical characteristics (table 2). Nevertheless, adjustment for NT-proBNP additional to clinical characteristics leads to non-significant results.

**Table 2:** Associations between sST2 and the primary (any cardiovascular event) and secondary endpoints (death or heart failure), with adjustment for clinical characteristics

	HR per twofold higher value	95% CI	P value
**Any cardiovascular event (n=225)**			
sST2 (univariable)	1.30	1.07 to 1.57	0.007
Adjusted for age and sex	1.47	1.19 to 1.81	<0.001
Adjusted for age, sex and creatinine	1.44	1.17 to 1.78	<0.001
Adjusted for age, sex and NT-proBNP	1.28	1.04 to 1.57	0.022
Adjusted for age, sex, NT-proBNP and creatinine	1.28	1.03 to 1.58	0.025
Adjusted for age, sex, rhythm and systemic ventricular function	1.46	1.19 to 1.79	<0.001
Adjusted for age, sex, congenital diagnosis*, NYHA class and cardiac medication	1.29	1.05 to 1.58	0.016
Full model†	1.28	1.05 to 1.59	0.017
Full model† and NT-proBNP	1.19	0.96 to 1.48	0.106
**Death or heart failure (n=69)**			
sST2 (univariable)	1.48	1.04 to 2.10	0.029
Adjusted for age and sex	2.23	1.50 to 3.30	<0.001
Adjusted for age, sex and creatinine	2.16	1.45 to 3.22	<0.001
Adjusted for sex, age and NT-proBNP	1.48	0.99 to 2.22	0.059
Adjusted for sex, age, NT-proBNP and creatinine	1.57	1.03 to 2.39	0.036
Adjusted for age, sex, rhythm and systemic ventricular function	2.13	1.46 to 3.12	<0.001
Adjusted for age, sex, congenital diagnosis*, NYHA class and cardiac medication	1.60	1.10 to 2.34	0.015

*Congenital diagnosis of arterial switch operation, aortic stenosis or aortic coarctation (0) versus tetralogy of Fallot, Rastelli, systemic right ventricle, univentricular heart or pulmonary arterial hypertension (1).

†Adjusted for age, sex, creatinine, sinus rhythm, systemic ventricular function, congenital diagnosis, NYHA class 2–3 and cardiac medication. Analysis including all covariates (full model) was not performed for the secondary endpoint due to insufficient statistical power.

NT-proBNP, N terminal pro-B-type natriuretic peptide; NYHA, New York Heart Association; sST2, soluble suppression of tumourigenicity-2.

Stratified analysis according to moderate and complex ACHD showed that sST2 was significantly associated with both the primary and secondary endpoint in complex ACHD. Moreover, sST2 remained significantly associated with the primary endpoint after full adjustment for clinical characteristics and NT-proBNP in complex ACHD. In moderate ACHD, sST2 yielded no prognostic value (table 3).

**Table 3:** Stratified analysis for the association between sST2 and the primary endpoint (any cardiovascular event) and secondary endpoints (death or heart failure) according moderate and complex adult congenital heart disease (ACHD)

	Moderate (n=266)			Complex (n=324)		
	HR*	95% CI	P value	HR*	95% CI	P value
**Any cardiovascular event**	**n=73**			**n=152**		
sST2 (univariable)	0.81	0.57 to 1.15	0.234	1.61	1.27 to 2.03	<0.001
Adjusted for age and sex	0.88	0.58 to 1.34	0.561	1.77	1.37 to 2.28	<0.001
Adjusted for age, sex and creatinine	0.89	0.59 to 1.35	0.591	1.73	1.34 to 2.23	<0.001
Adjusted for age, sex and NT-proBNP	0.83	0.56 to 1.24	0.368	1.53	1.19 to 1.97	<0.001
Adjusted for age, sex, NT-proBNP and creatinine	0.83	0.56 to 1.24	0.363	1.53	1.18 to 1.99	0.001
Adjusted for age, sex, rhythm and systemic ventricular function	0.86	0.56 to 1.31	0.478	1.76	1.37 to 2.25	<0.001
Adjusted for age, sex, NYHA class and cardiac medication	0.90	0.59 to 1.37	0.620	1.40	1.10 to 1.78	0.007
Adjusted for full model†	0.86	0.56 to 1.30	0.470	1.37	1.07–1.76	0.013
Adjusted for full model† and NT-proBNP	0.80	0.54 to 1.21	0.294	1.31	1.01 to 1.69	0.043
**Death or heart failure**	**n=15**			**n=54**		
sST2 (univariable)	0.52	0.24 to 1.14	0.100	1.87	1.25 to 2.80	0.002
Adjusted for age and sex	–	–	–	2.68	1.75 to 4.11	<0.001
Adjusted for age, sex and creatinine	–	–	–	2.53	1.62 to 3.95	<0.001
Adjusted for age, sex and NT-proBNP	–	–	–	1.88	1.20 to 2.95	0.006
Adjusted for age, sex, NT-proBNP and creatinine	–	–	–	1.93	1.20 to 3.12	0.007

Moderate ACHD: arterial switch operation, aortic stenosis or aortic coarctation.

Complex ACHD: tetralogy of Fallot, Rastelli, systemic right ventricle, univentricular heart or pulmonary arterial hypertension. Due to only a limited number of events, no further adjustment for clinical characteristics was performed regarding the secondary endpoint.

*HRs are expressed per twofold higher sST2 level.

†Adjusted for age, sex, creatinine, sinus rhythm, systemic ventricular function, NYHA class 2–3 and cardiac medication.

NT-proBNP, N terminal pro-B-type natriuretic peptide; NYHA, New York Heart Association; sST2, soluble suppression of tumourigenicity-2.

### Sex-specific differences of sST2

Sex and sST2 showed a significant interaction; therefore, we performed a stratified post hoc analysis by sex. Women were older, had a higher heart rate, lower systolic blood pressure and higher NT-proBNP levels than men (online supplementary file 5). Survival analysis showed that women in the fourth quartile of sST2 (sST2 >25.2 ng/mL) were at higher risk of endpoints. In men, there was no significant difference in endpoint-free survival among sST2 quartiles (online supplementary file 6). Analysis of continuous levels showed that in women sST2 was strongly associated with the endpoints, independent of age, creatinine and NT-proBNP. In men, these associations were absent (table 4).

**Table 4:** Stratified analysis for the association between sST2 and the primary endpoint (any cardiovascular event) and secondary endpoint (death or heart failure) according to women and men

	Women			Men		
	HR*	95% CI	P value	HR*	95% CI	P value
**Any cardiovascular event**	**n=96**			**n=129**		
sST2 (univariable)	1.80	1.30 to 2.49	<0.001	1.19	0.90 to 1.56	0.223
Adjusted for age	1.72	1.22 to 2.44	0.002	1.30	0.99 to 1.71	0.063
Adjusted for age and creatinine	1.71	1.22 to 2.41	0.002	1.28	0.97 to 1.69	0.080
Adjusted for age and NT-proBNP	1.49	1.04 to 2.12	0.029	1.16	0.89 to 1.51	0.274
Adjusted for age, NT-proBNP and creatinine	1.48	1.04 to 2.11	0.029	1.18	0.89 to 1.55	0.248
Adjusted for age, rhythm and systemic ventricular function	1.67	1.20 to 2.33	0.002	1.28	0.98 to 1.68	0.071
Adjusted for age, congenital diagnosis†, NYHA class and cardiac medication	1.23	0.87 to 1.75	0.240	1.30	0.99 to 1.70	0.056
Adjusted for full model‡	1.26	0.89 to 1.79	0.190	1.28	0.97 to 1.69	0.076
Adjusted for full model‡ and NT-proBNP	1.17	0.81 to 1.69	0.407	1.18	0.89 to 1.56	0.250
**Death or heart failure**	**n=40**			**n=29**		
sST2 (univariable)	2.72	1.67 to 4.44	<0.001	1.47	0.81 to 2.65	0.201
Adjusted for age	2.68	1.55 to 4.63	<0.001	1.83	1.03 to 3.23	0.039
Adjusted for age and creatinine	2.68	1.55 to 4.62	<0.001	1.61	0.88 to 2.94	0.125
Adjusted for age and NT-proBNP	2.03	1.12 to 3.69	0.020	1.18	0.69 to 2.00	0.547
Adjusted for age, NT-proBNP and creatinine	2.04	1.12 to 3.71	0.020	– §	–	–

Analysis including all covariates (full model) was not performed for the secondary endpoint due to insufficient statistical power.

*HRs are expressed per twofold increase in sST2 level. P value of interaction between sex and sST2=0.047 (primary endpoint) and p=0.104 for secondary endpoint.

†Congenital diagnosis of arterial switch operation, aortic stenosis or aortic coarctation (0) versus tetralogy of Fallot, Rastelli, systemic right ventricle, univentricular heart or pulmonary arterial hypertension (1).

‡Adjusted for age, creatinine, sinus rhythm, systemic ventricular function, congenital diagnosis, NYHA class 2–3 and cardiac medication.

§Insufficient statistical power to perform analysis.

NT-proBNP, N terminal pro-B-type natriuretic peptide; NYHA, New York Heart Association; sST2, soluble suppression of tumourigenicity-2.

## Discussion

This study investigated the prognostic value of sST2 in a large prospective cohort of adults with congenital heart disease. Higher levels of sST2 were found in patients with more complex congenital heart disease. Moreover, levels of sST2 were significantly associated with cardiovascular events, even independent of the established biomarker NT-proBNP. Diagnosis-specific analysis showed a significant prognostic value for sST2 in complex ACHD independent of clinical characteristics and NT-proBNP, while in moderate ACHD, sST2 yielded no prognostic value. We also revealed important sex-specific differences of sST2; both in healthy controls and patients with ACHD, sST2 was lower in woman throughout all age categories. In addition, whereas sST2 was significantly associated with cardiovascular events in woman, this association was absent in men.

### Previous reports

As sST2 has been investigated extensively in HF, it has only been described once in ACHD. Laqqan *et al* 7 investigated sST2 in complex ACHD patients with a wide age range (12–70 years) and identified sST2 as strong prognostic biomarker. The prognostic value of sST2 in patients with chronic and acute HF has been established firmly and is described by Aimo *et al* in two meta-analysis. In both populations, sST2 aids the risk stratification.^5 6^ In patients with acute HF, sST2 levels rose in the period prior to readmission for HF or death and serial sST2 measurements better predicted adverse outcomes compared with a single measurement, independent of serial NT-proBNP measurements.^14^ Finally, another study showed that higher levels of sST2 were predictive of survival after transcathether aortic valve implantation in patients with aortic stenosis.^15^

### Pathophysiology of sST2

Soluble ST2 is the circulating form of the transmembrane ST2 ligand, which is the receptor for interleukin-33. sST2 acts as a decoy receptor for interleukin-33 and therefore increased sST2 levels undermine the effects of the interleukin-33/ST2 ligand interaction.^16 17^ The interleukin-33/ST2 ligand signalling plays an important role in protecting the myocardium against maladaptive hypertrophy and fibrosis. As sST2 blocks this IL-33/ST2 ligand complex, these cardiac protective effects will be abolished and ventricular failure may develop.^18^ In our study, sST2 and NT-proBNP levels were not correlated in complex ACHD. In the first and only study previously investigating sST2 in patients with complex ACHD, only a very weak correlation was found between sST2 and NT-proBNP (r=0.29, p<0.001).^7^ This may suggest that sST2 is involved in another pathophysiological pathway than NT-proBNP regarding myocardial adaptation and dysfunction.

Besides the association with myocardial stress, sST2 is also known for its relation with inflammatory and immune processes.^17^ sST2 has been investigated as inflammatory marker in numerous diseases such as asthma, chronic obstructive pulmonary disease, collagen vascular diseases, trauma and sepsis.^19^ Although it is unlikely that sST2 levels were influenced by inflammatory processes in our patients, we cannot preclude that sST2 levels may have been influenced by other unknown processes.

### sST2 in healthy individuals

Reference values established in this study were higher for both sexes than reference values described in previous studies using the same ST2 assay.^20–22^ However, median/mean sST2 levels were comparable with most values reported in literature.^21 22^ A reason for the high reference values may be the relatively limited number of healthy volunteers and therefore the stronger influence of outliers. These high reference values could explain the relatively low number of ACHD patients with an elevated level of sST2 in our study. Identifying patients in our study with elevated sST2 levels based on reference values from the Framingham Heart Study resulted in 19 women (7.7%) (>33.2 ng/mL) and 38 men (11.1%) (>47.6 ng/mL) with an elevated sST2. This would mean that in 9.7% of the patients sST2 was elevated in contrast to the 3.7% we identified.

Although sST2 levels measured in patients with ACHD seemed comparable with the ones found in the healthy volunteers, it is unclear whether sST2 has the same prognostic value in healthy volunteers as in patients with ACHD. A study investigating sST2 in a Finnish healthy cohort showed that sST2 did not improve long-term prediction of cardiovascular events.^23^ In contrast, the Framingham Heart Study found that higher sST2 preceded cardiac adverse events during a mean follow-up of 11.3 years in the general population.^24^

### Sex-specific differences of sST2

Our study in healthy volunteers found that sST2 levels were significantly lower among women than men; this finding is consistent with previous studies.^21 22^ Normal ranges studied in the Framingham Heart Study found that both sex and age are important determinants of sST2 levels. They described that women taking oestrogen replacement therapy had lower levels of sST2,^13^ suggesting that hormone release may explain the sex-difference in sST2 levels. However, another study investigating the association between ssST2 and hormones in healthy men and women did not find an independent association.^25^ Currently, there has been no clear explanation for these sex-differences and whether sST2 synthesis or secretion is under hormonal control or not.

The patients in our study were much younger compared with the general HF population. Most women presumably are premenopausal and may use hormone replacement as anticonception. This could be an explanation for the interaction between sST2 and sex that we found. Unfortunately, we had no data on hormone levels. Further research is warranted to elucidate the conflicting results on hormonal influences and sST2 in premenopausal women. This is of particular interest in the ACHD population, which is characterised by a relatively young age.

There was no significant association between sST2 and the primary endpoint in men, and it seemed that the height of sST2 and the associated risk reached a ceiling, creating a situation in which higher sST2 levels do not reflect higher risks. Although there are no data to support this, a possible explanation might be that men have more fluctuating and extreme levels of sST2. Hormonal influences in women might cause more stable and less extreme sST2 levels leading to a more stable prognostic effect of sST2 over time in women.

### Clinical perspectives

sST2 may specifically be useful as prognostic biomarker in stable adults with complex congenital heart disease. Therefore, an sST2 measurement could be considered in these patients, besides an NT-proBNP measurement. Our study was conducted in clinically stable adult patients, and it would be interesting to know whether an increase in sST2 over time reflects clinical worsening. Serial measurements of NT-proBNP have been investigated in ACHD, and increased NT-proBNP levels were found before the occurrence of cardiovascular events.^26^ sST2 has a narrower biological variation in comparison to NT-proBNP,^27^ which is an advantage when measuring a biomarker repeatedly. For this reason, sST2 may be a very suitable biomarker in clinical practice to monitor patients over time. Patients with ACHD are characterised by a high need for reinterventions; 23% of the patients in our cohort needed a reintervention during follow-up. A biomarker that could aid with the right timing of reinterventions is therefore highly desirable in this population.

### Limitations

Serum samples were stored for a duration by −80°C before sST2 was measured. It is unknown whether sST2 levels are affected by this long storage period; however, one study showed that sST2 is stable in plasma samples for a maximum storage period of 1.5 years at −80°C.^28^ In our study, there was no correlation found between storage time and sST2 levels.

A heterogeneous group of diagnoses are included in this ACHD cohort. sST2 levels were higher in the patients with complex ACHD, in which sST2 yielded a strong prognostic value. In contrast, no prognostic value of sST2 was found in moderate ACHD. Unfortunately, diagnoses-specific subgroup analysis were restricted by the limited sample size within each diagnosis group. Patients with isolated repaired atrial or ventricular septal defect were not included in this study due to the expected low number of events. This should be kept in mind when extrapolating the results to other ACHD cohorts. sST2 is a relatively expensive biomarker and currently not available in standard laboratories. Implementing sST2 in the current risk stratification is therefore challenging. However, over the past years, sST2 has increasingly been used in patients with HF, potentially expanding the availability of the ST2 assay in the future.

## Conclusion

sST2 is significantly associated with adverse cardiovascular events in patients with complex ACHD, independent of the conventional biomarker NT-proBNP. Sex-specific analyses revealed a strong association between sST2 and cardiovascular events in women; however, this association was absent in men. These sex differences of sST2 need further clarification. Nonetheless, sST2 seems to be a potential new prognostic biomarker in patients with ACHD.

**Key messages**

**What is already known on this subject?**

- Soluble suppression of tumourigenicity-2 (sST2) is emerging as new prognostic biomarker in patients with heart failure. Only very limited data are available on sST2 in adults with congenital heart disease (ACHD), while this population is characterised by a high incidence of heart failure.

**What might this study add?**

- In patients with ACHD who are seen at the outpatient clinic, higher sST2 levels are associated with an increased risk of cardiovascular events, independent of N terminal pro-B type brain natriuretic peptide (NT-proBNP) levels. This association was specifically present in women and adults with complex congenital heart disease.

**How might this impact on clinical practice?**

- sST2 can be a potential new prognostic biomarker for risk stratification in ACHD. Besides NT-proBNP, an sST2 measurement should be considered mainly in patients with complex ACHD. Sex-specific cut-off levels should be applied.
