# Supplementary material for: Prognostic value of soluble ST2 in adults with congenital heart disease
Source: Heart. 2019 Jan 30;105(13):999–1006. doi: 10.1136/heartjnl-2018-314168 (PMC6582725; doi:10.1136/heartjnl-2018-314168)
Supplement: Supplementary data [file heartjnl-2018-314168supp001.pdf]

The prognostic value of soluble ST2 in adults with congenital heart disease

Authors:

Laurie W. Geenen, BSc, Vivan J.M. Baggen, MD, Annemien E. van den Bosch, MD, PhD , Jannet A. Eindhoven, MD, PhD, Judith A.A.E Cuypers, MD, PhD, Maarten Witsenburg , MD, PhD, Eric Boersma, MSc, PhD Jolien W. Roos-Hesselink, MD, PhD

SUPPLEMENTARY MATERIAL

List of files **Page**

Supplementary File 1. Flowchart of the patient selection process……………………………...............1

Supplementary File 2. Scatterplot for the correlation between sST2 and NT-proBNP………………2

Supplementary File 3. sST2 levels according to percentiles found in healthy volunteers and ACHD patients, stratified according to women and men……………………………………………….………3

Supplementary File 4. Reproducibility of the ST2 assay in healthy volunteers shown by a Bland-Altman plot..……………………………………………………...4

Supplementary File 5. Baseline characteristics of adult congenital heart patients stratified according to sex……………..…………………………………………………………………………………5

Supplementary File 6. Survival curves according to the quartile distribution of sST2 for the primary and secondary endpoint, shown separately for men and women..……………………………………..7

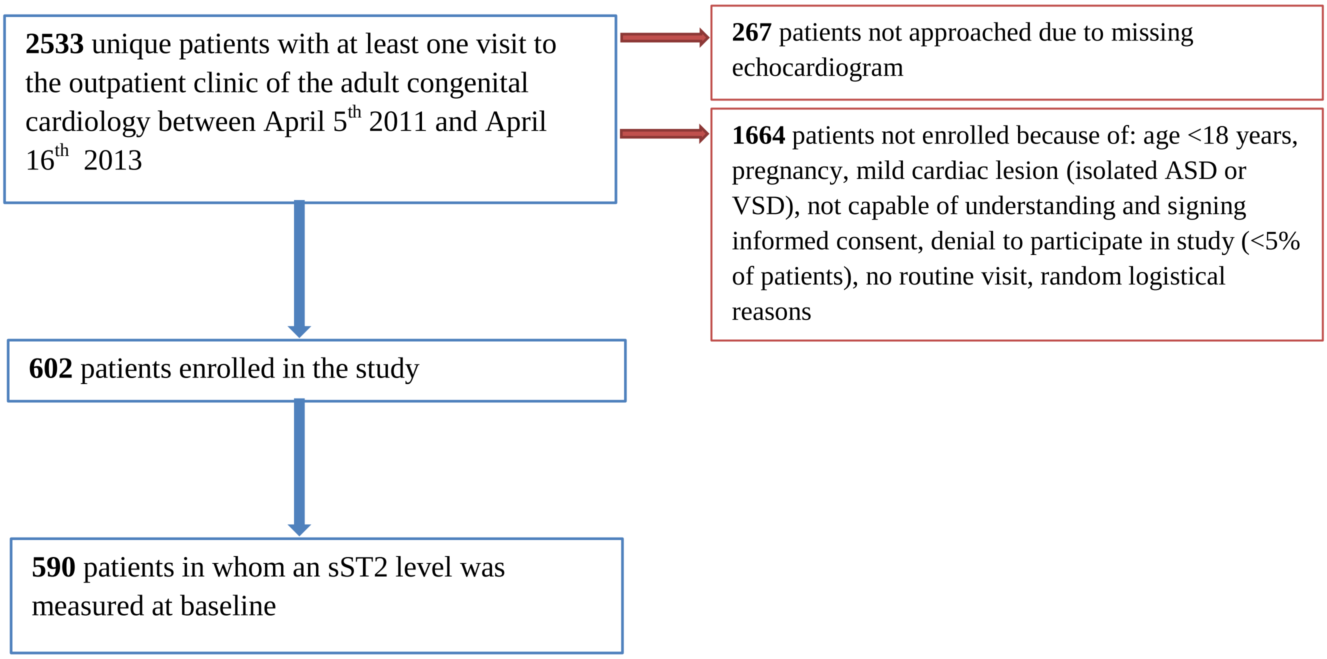

Supplementary File 1. Flowchart of the patient selection process.

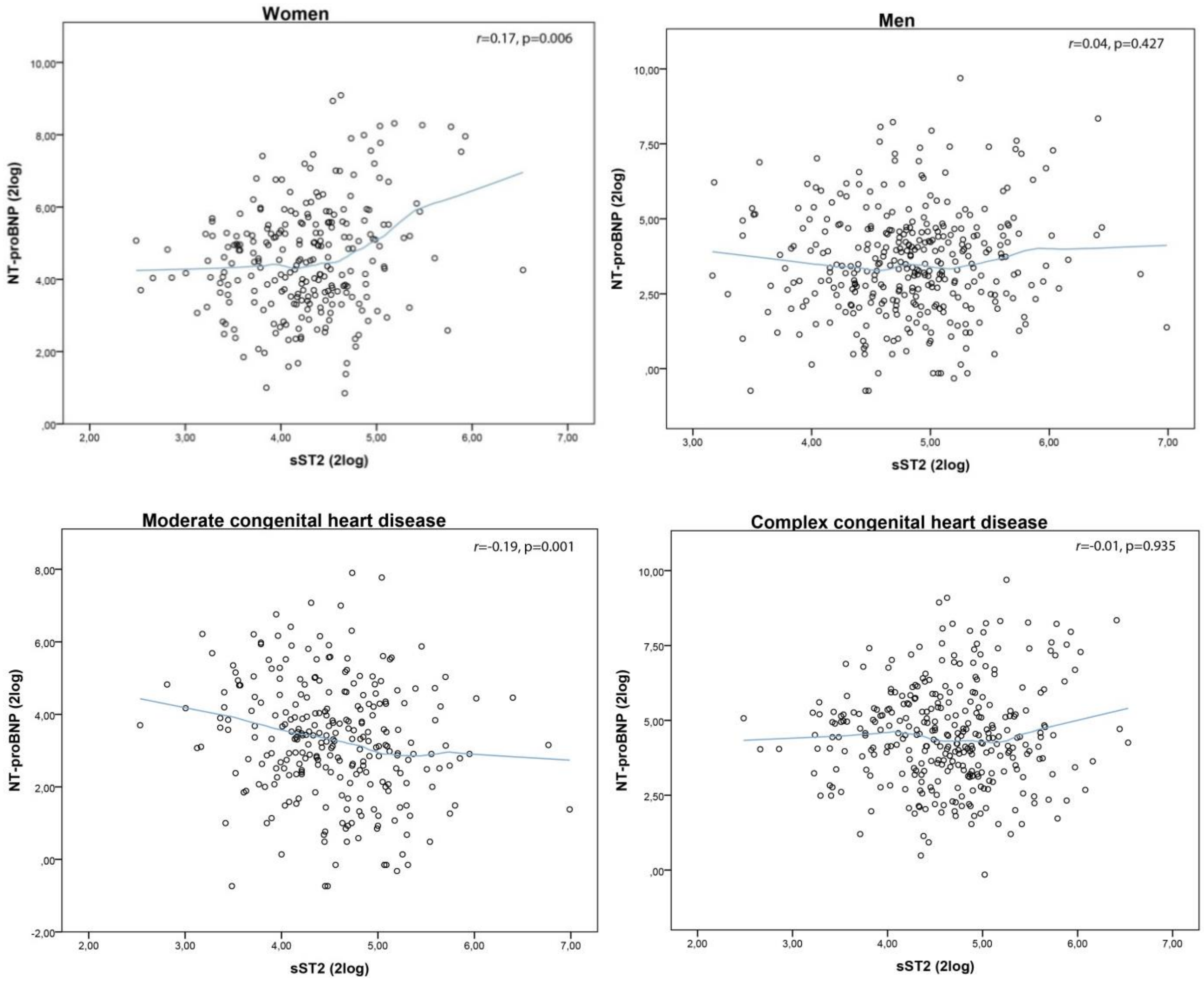

Supplementary File2. Scatterplot showing the correlation between sST2 and NT-proBNP levels, stratified according to women and men and according to moderate and complex congenital heart disease.

The blue line represents the Loess curve for the association between the data points.

Moderate congenital heart disease included: arterial switch operation, aortic stenosis or aortic coarctation

Complex congenital heart disease included: Tetralogy of Fallot, Rastelli, systemic right ventricle, univentricular heart or pulmonary arterial hypertension

sST2 percentiles →	**25th**	**50th**	**75th**	**95th**	**97.5th**	***Cut-off****
			**Women** sST2 (ng/mL)			
**Healthy volunteers**	13.9	20.1	25.0	40.4	54.9	***44.5***
**ACHD patients**	14.5	19.5	25.2	37.8	47.9	
			**Men** sST2 (ng/mL)			
**Healthy volunteers**	19.4	25.5	32.8	42.4	60.4	***55.9***
**ACHD patients**	21.9	28.5	36.7	55.2	65.1	

Supplementary File 3. sST2 values according to percentiles found in healthy volunteers and ACHD patients, stratified according to women and men.

* Cut-off to define elevated levels of sST2, calculated based on the following formula: mean + 1.96 SD (on the 2log scale)

Abbreviations: ACHD= adult congenital heart disease, sST2= soluble suppression of tumorigenecity-2

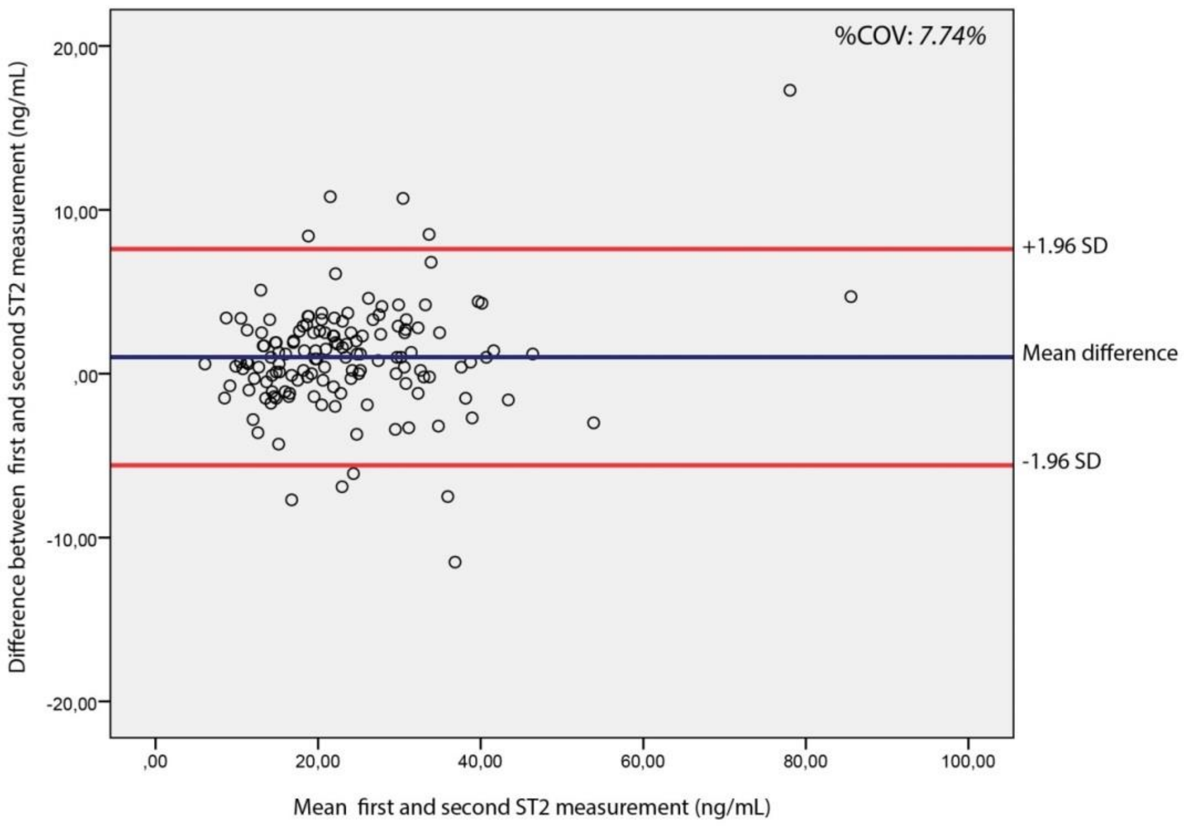

Supplementary File 4. Bland-Altman plot showing the reproducibility of the ST2 assay in healthy individuals.

The blue line represents the mean difference of the two sST2 measurements. The red lines indicate the limits of agreement. COV=coefficient of variation, expressed as percentage. SD= standard deviation.

	Women (n=248)	Men (n=342)	P-value
**Clinical characteristics**			
Age, years	33.9 [25.1-44.8]	31.7 [24.2-40.1]	0.033
Surgical repair, n (%)	222 (90)	316 (92)	
Age at surgical repair, years	4.8 [1.0-11.6]	3.1 [0.7-12.2]	0.145
Congenital diagnosis, complex*, n (%)	133 (54)	191 (56)	0.286
Cardiac medication use†, n (%)	93 (38)	118 (35)	0.562
Body mass index, kg/m2	25.1 ± 5.0	24.5 ± 3.9	0.09
Heart rate, beats/minute	75 ± 14	73 ± 13	0.036
Systolic blood pressure, mmHg	124 ± 18	128 ± 14	0.011
O2 saturation <90%, n (%)	12 (5)	5 (2)	0.016
NYHA class, II or III , n (%)	32 (13)	29 (9)	0.082
**Electrocardiography**			
Rhythm n (%)			0.005
Sinus rhythm	194 (78)	277 (81)	
Paced rhythm	18 (7)	26 (8)	
Other	36 (15)	39 (11)	
QRS duration, ms	106 [94-136]	116 [105-142]	<0.001
**Echocardiography**			
Left atrial volume, mL/m2	20.8 [16.1-29.9]	20.5 [15.1-28.0]	0.508
Left ventricular end-diastolic volume, mL/m2‡	27.2 ± 4.0	25.3 ± 3.4	<0.001
Left ventricular ejection fraction, %‡	56.6 ± 7.4	55.6 ± 8.0	0.204
Right ventricular end diastolic annulus diameter, mm	39.7 ± 7.6	43.98 ± 8.2	<0.001
Right ventricular fractional area change, %	39.9 ± 10.8	37.1 ± 11.5	0.020
Systemic ventricular function, n (%)			0.04
Normal	128 (52%)	168 (49)	
Mildly impaired	95 (38)	112 (33	
Moderately impaired	21 (9)	48 (14)	
Severely impaired	4 (2)	14 (4)	
E/A ratio	1.7 ± 0.7	1.6 ± 0.6	0.012
E' wave, m/s	7.8 ± 2.5	8.5 ± 2.6	<0.001
E/E' ratio	13.0 ± 5.4	10.6 ± 4.5	<0.001
**Laboratory results**			
Creatinine, µmol/L	69.9 ± 18.8	82.0 ± 15.2	<0.001
NT-proBNP, pmol/L	22.8 [11.5-45.8]	10.8 [5.1-24.3]	<0.001

Supplementary File 5. Baseline characteristics of the adult congenital heart disease cohort, stratified according to sex.

Differences between women and men were assessed and expressed by the p-value. A t-test was performed for normal distributed continuous variables otherwise the Mann-Whitney U test was used. For categorical variables, the chi squared test or Fisher Exact test was used, as appropriate.

[*]Congenital diagnosis of arterial switch operation, aortic stenosis or aortic coarctation (0) versus Tetralogy of Fallot, Rastelli, systemic right ventricle, univentricular heart or pulmonary arterial hypertension (1) [†] Beta-blocker (=90 ,15 %), ACE inhibitor (n=88 ,15 %), diuretic (n=71,12 %), antiarrhythmic (n=53 ,9 %) angiotensin receptor blocker (n=36, 6 %) [‡] Left-sided volumes were not measured in patients with a systemic right ventricle, univentricular heart, pulmonary hypertension or a poor acoustic window.

Abbreviations: NT-proBNP= N-terminal pro-B type brain natriuretic peptide, NYHA= New York Heart Association, sST2= soluble suppression of tumorigenicity-2

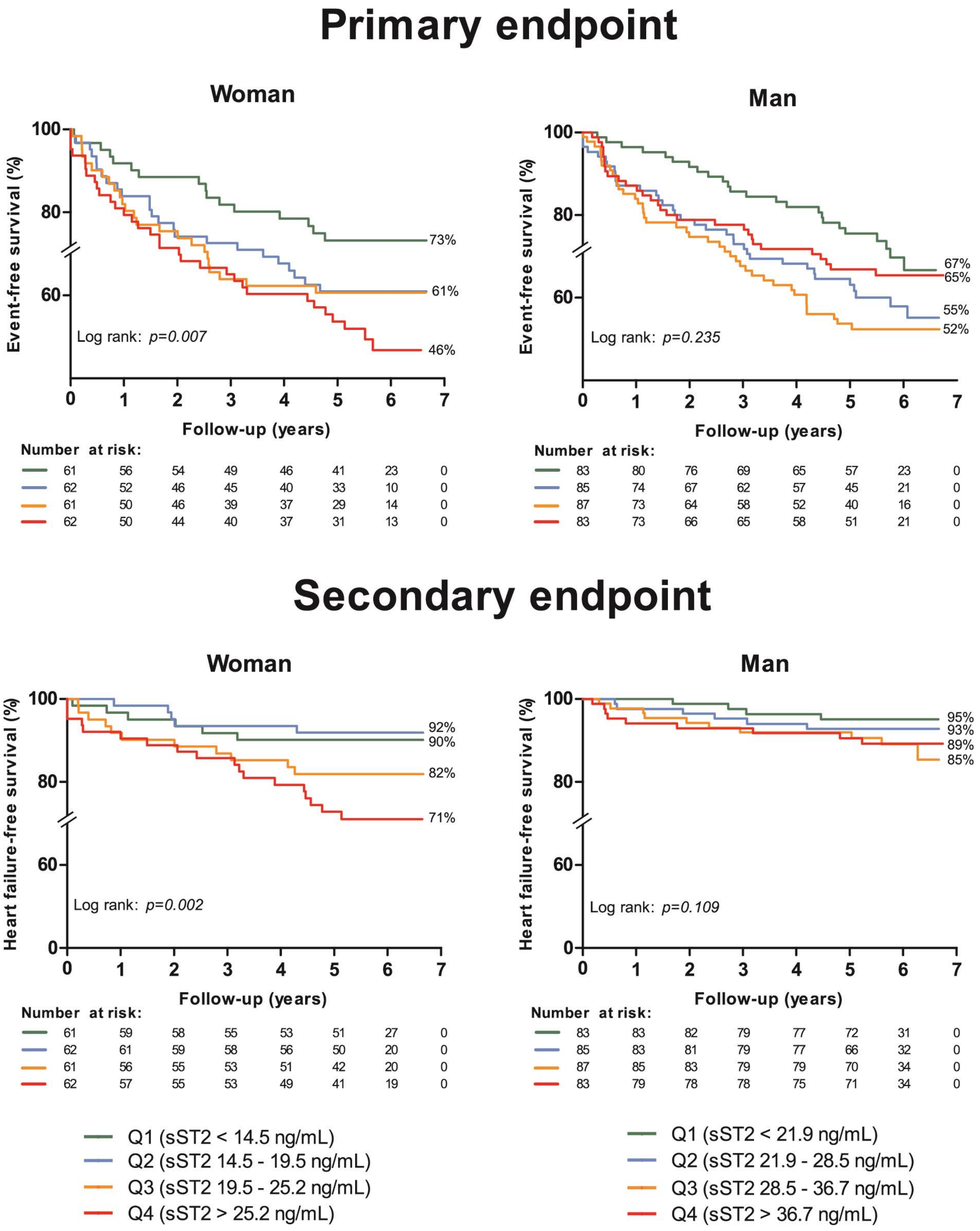

Supplementary File 6. Kaplan-Meier curve showing the event-free (primary endpoint) and heart failure-free survival (secondary endpoint) according to the quartile distribution of sST2 for women and men separately in adults with congenital heart disease.

Sex-specific quartile distributions of sST2 are specified in the legend.

Abbreviations: Q1= quartile 1, Q2= quartile 2, Q3= quartile 3, Q4= quartile 4, sST2= soluble suppression of tumorigenicity-2.
